# Chromatin Remodeler RSF1 as an Oncogenic Driver and Therapeutic Target in Esophageal Squamous Cell Carcinoma

**DOI:** 10.3390/cells14161262

**Published:** 2025-08-15

**Authors:** Zhenhua Du, Zhili Jia, Yao Lin, Xudong Zhao, Gengsheng Cao, Hengbin Wang

**Affiliations:** 1 School of Life Sciences, Henan University, Kaifeng 475004, China; hzdw0324@163.com (Z.D.); chilejia@163.com (Z.J.); linyusen@henu.edu.cn (Y.L.); zhaoxudong@connect.hku.hk (X.Z.); 2 Department of Obstetrics and Gynecology, Li Ka Shing Faculty of Medicine, The University ofHong Kong, Hong Kong 999077, China; 3 Department of Internal Medicine, Division of Hematology, Oncology and Palliative Care, Massey Comprehensive Cancer Center, Virginia Commonwealth University, Richmond, VA 23298, USA

**Keywords:** RSF1, esophageal squamous cell carcinoma, cell cycle, apoptosis

## Abstract

Esophageal squamous cell carcinoma (ESCC) is a prevalent malignancy, ranking eleventh in incidence and seventh in mortality globally. Remodeling and Spacing factor 1 (RSF1), a chromatin remodeling factor, is frequently overexpressed in various tumors and correlates with poor prognosis. This study, combining public database analysis and clinical sample validation, reveals significantly elevated RSF1 expression in ESCC tumor tissues, confirmed further in an ESCC orthotopic model. Functional assays show that RSF1 knockout (KO) significantly inhibits ESCC cell proliferation, migration, invasion, and in vivo tumor growth, while reintroducing RSF1 restores its oncogenic effects. Proteomic analysis highlights that RSF1 KO disrupts pathways associated with cell cycle control, apoptosis, and focal adhesion. Experimentally, RSF1 KO induces apoptosis and G2/M arrest, establishing its essential role in ESCC progression. Collectively, these findings establish RSF1 as an oncogenic driver and a promising therapeutic target in ESCC.

## 1. Introduction

Esophageal squamous cell carcinoma (ESCC) is a highly prevalent malignancy, particularly in China [1,2,3,4,5]. Despite advances in comprehensive treatment strategies, the 5-year survival rate for ESCC remains dismally low [6,7,8]. The disease follows a prolonged and stepwise progression from mild, moderate, and severe atypical hyperplasia, to carcinoma in situ, invasive carcinoma, and, eventually, metastatic disease [9,10]. This multistage transformation is orchestrated by a complex interplay of genetic and molecular factors [11,12,13]. Therefore, identifying novel key drivers and biomarkers is critical for improving early detection, prognostication, and developing targeted therapies.

Remodeling and Spacing Factor 1 (RSF1), a core subunit of the chromatin remodeling RSF complex, functions in nucleosome repositioning and transcriptional regulation [14,15,16,17]. RSF1 is frequently overexpressed across diverse human cancers and consistently correlates with aggressive tumor behavior and poor clinical outcomes [18,19]. Mechanistic studies link RSF1 to cancer progression through various pathways, including enhancing NF-κB-mediated chemoresistance, maintaining genomic stability, and facilitating DNA damage repair [14,20,21,22,23]. These multifaceted roles underscore RSF1′s potential as a significant oncoprotein.

However, the specific contribution of RSF1 to ESCC pathogenesis remains poorly defined. Its expression pattern, functional significance, and downstream mechanisms in ESCC are largely unexplored, creating a critical knowledge gap in understanding ESCC development and identifying actionable targets.

To address this, we undertook a comprehensive study to elucidate the role of RSF1 in ESCC. Combining analysis of public databases, validation in clinical specimens, and rigorous in vitro and in vivo functional assays, we demonstrate that RSF1 is significantly upregulated in ESCC tumors and cell lines. Genetic ablation of RSF1 potently suppressed ESCC cell proliferation, migration, invasion, and tumor growth in vivo, while reconstitution of RSF1 rescued these oncogenic phenotypes. Mechanistically, proteomic profiling and functional validation revealed that RSF1 depletion induces apoptosis, at least partly by shifting the Bcl-2/BAX balance towards pro-apoptotic signaling, and triggers G2/M cell cycle arrest. Our findings establish RSF1 as a crucial oncogenic driver in ESCC and validate its potential as a therapeutic target.

## 2. Materials and Methods

### 2.1. Cell Lines and Culture Conditions

KYSE 30, KYSE 70, KYSE 140, KYSE 150, KYSE 410, KYSE 450, KYSE 510, KYSE 520, and EC9706 esophageal squamous cell carcinoma (ESCC) cell lines were obtained from the American Type Culture Collection (ATCC). All cell lines were maintained in RPMI 1640 medium (Corning, NY, USA, cat# 10-040-CV) supplemented with 10% fetal bovine serum (FBS; Corning, NY, USA, cat# 35-082-CV) and 1% penicillin–streptomycin (Beyotime Biotechnology, Shanghai, China, cat# C0222) at 37 °C in a humidified incubator with 5% CO_2_. HEK293T cells were cultured in DMEM (Corning, NY, USA, cat# 10-013-CV) supplemented with 10% FBS (Corning, NY, USA, cat# 35-082-CV) and 1% penicillin–streptomycin (Beyotime Biotechnology, Shanghai, China, cat# C0222) under the same incubation conditions.

### 2.2. Transfection and Lentivirus Production

sgRNAs were designed using the CRISPOR online platform (https://crispor.gi.ucsc.edu/). The oligonucleotide sequences were as follows:

sgRNA1:

Sense: 5′-TTAAGTACCAAAAGAATTGG-3′;

Antisense: 5′-CCAATTCTTTTGGTACTTAA-3′.

sgRNA2:

Sense: 5′-ATCTGTTACTGCAGACAGAT-3′;

Antisense: 5′-ATCTGTCTGCAGTAACAGAT-3′.

Lentiviral particles were packaged in HEK293T cells following standard protocols. Target cells were transduced with lentivirus for 48 h, followed by selection with 1.5 μg/mL puromycin. Puromycin-resistant colonies were expanded and validated for transgene expression via immunoblotting.

For RSF1 overexpression, the full-length human RSF1 coding sequence was cloned into the pLVX-IRES-Puro vector via EcoRI (Thermo Fisher Scientific; Thermo Scientific, Rockford, IL, USA, cat# FD0274) and BamHI (Thermo Fisher Scientific; Thermo Scientific, Rockford, IL, USA, cat# FD0054) restriction sites.

### 2.3. CCK8 Assay

Cell proliferation was evaluated using the Cell Counting Kit (CCK-8; C0037, Beyotime Biotechnology, Shanghai, China) assay in wild-type (WT) and RSF1-knockout (KO) ESCC cell lines (KYSE450 and EC9706). Cells were seeded in 96-well plates at a density of 1.5 × 10^3^ cells/well and incubated at 37 °C in a humidified atmosphere containing 5% CO_2_. After cell attachment, 10 μL of CCK-8 reagent was added to each well and incubated for 2 h at 37 °C in a humidified atmosphere containing 5% CO_2_. The optical density (OD) at 450 nm was measured using a Tecan Infinite M PLEX microplate reader. This reading served as the Day 0 baseline. Subsequent OD measurements were taken every 24 h for five additional days, resulting in a total of six time points. Growth curves were piloted from the collected data.

### 2.4. Colony Formation Assays

Colony-forming ability was evaluated in wild-type (WT) and RSF1-knockout (KO) ESCC cell lines (KYSE450 and EC9706) using the following protocol. Briefly, cells were seeded in 6-well plates at a density of 750 cells/well and cultured for 7–14 days to allow colony formation. At the end of the incubation period, the medium was aspirated, and cells were washed three times with PBS, fixed with 4% paraformaldehyde for 30 min at room temperature, and stained with 0.1% crystal violet for 20 min. Excess staining was removed by gently rinsing the plate with running tap water. Plates were then air-dried overnight and imaged to assess colony formation.

### 2.5. EdU Assay

DNA synthesis was assessed using EdU (5-ethynyl-2′-deoxyuridine) incorporation. Wild-type and RSF1-knockout ESCC cell lines (KYSE450 and EC9706) were seeded into 96-well plates and cultured to logarithmic growth phase. Cells were then incubated with medium containing 50 μM EdU for 2 h at 37 °C in a humidified incubator with 5% CO_2_. After incubation, the medium was removed, and cells were washed three times with PBS. Cells were then fixed with 4% paraformaldehyde for 30 min at room temperature, followed by three additional washes. To quench residual fixative, 50 μL of 2 mg/mL glycine solution was added for 5 min, and then discarded. Permeabilization was performed using 100 μL of PBS containing 0.5% Triton X-100 for 10 min, followed by PBS wash. EdU detection was carried out using Apollo staining reaction solution (RiboBio, Guangzhou, China, Cat# C10310-3), according to the manufacturer’s instructions. Cells were incubated at room temperature in the dark for 30 min, after which the staining solution was discarded and cells were washed three times with PBS containing 0.5% Triton X-100. Nuclei were counterstained with Hoechst 33342 for 30 min in the dark at room temperature, followed by three PBS washes. Antifade mounting medium was applied before imaging. Fluorescent images were acquired using an inverted fluorescence microscope, and cell proliferation was quantified using Zeiss image analysis software (ZEN Lite 2012, Germany).

### 2.6. Wound-Healing Assay

Cell migration was assessed using a wound-healing assay. Wild-type and RSF1-knockout ESCC cell lines (KYSE450 and EC9706) were seeded into 6-well plates and cultured under standard conditions until a confluent monolayer was reached. A reference line was drawn on the bottom of each well using a sterile marker to standardize the scratch position. A straight wound was created perpendicular to the reference line using a sterile 10 μL pipette tip. A new tip was used for each well to ensure consistency and prevent cross-contamination. Detached cells were removed by gently rinsing each well with PBS. Cells were then cultured in low-serum medium containing 2% FBS to minimize proliferation during the assay period. An initial image of the wound area was captured (T = 0 h) using an inverted phase contrast microscope, and at least the predefined areas were imaged in each well. Cells were incubated at 37 °C in a humidified incubator with 5% CO_2_ for 24 h. At T = 24 h, the same wound regions were re-imaged. Wound width was measured using ZEN imaging software (ZEN Lite 2012, Germany), and the wound-healing rate was calculated as the following formula:

Wound-healing rate (%) = [(average width at T = 0 h) − (average width at T = 24 h)]/(average width at T = 0 h) × 100.

### 2.7. Transwell Migration and Invasion Assays

Cell migration was measured using a Transwell insert (Falcon, Franklin Lakes, NJ, USA, Cat#. 353097). Wild-type and RSF1-knockout ESCC cells (KYSE450 and EC9706) were harvested, trypsinized, and resuspended in serum-free medium at 4 × 10^5^ cells/mL.

A total of 200 μL of cell suspension was seeded in the upper chamber; the lower chamber contained 600 μL of medium containing 15% fetal bovine serum (FBS) as a chemoattractant. After incubation at 37 °C in a humidified incubator with 5% CO_2_ for 24 h, non-migrated cells were removed from the upper membrane surface. Cells on the lower surface were fixed with 4% paraformaldehyde, stained with 0.1% crystal violet, and counted under an inverted microscope in five random fields per insert.

For invasion assays, Transwell chambers were coated with Matrigel (Corning, NY, USA). Matrigel was thawed overnight at 4 °C, and all materials were pre-chilled. Diluted Matrigel was applied to the upper chamber and polymerized at 37 °C for 2 h. Residual fluid was removed, and the layer was hydrated with serum-free medium for 30 min. Cell seeding, incubation, and subsequent steps followed the migration protocol.

### 2.8. Cell Apoptosis Analysis

Apoptosis was evaluated using the Annexin V-APC/PI Apoptosis Detection Kit (Multi Sciences, Hangzhou, China, Cat# AP107). Following treatment, cells were harvested, washed with cold PBS, and resuspended in binding buffer. Annexin V-APC (5 μL) and propidium iodide (10 μL) were added, and samples were incubated for 5 min at room temperature in the dark. Apoptotic cells were analyzed using a flow cytometer (CytoFLEX, Beckman Coulter, Beckman Coulter, Brea, CA, USA), and data were processed using FlowJo software (version 10.8.1).

### 2.9. Cell Cycle Analysis

Cell cycle distribution was measured using a cell cycle detection kit (Multi Sciences, Hangzhou, China, Cat# CCS012). After treatment, 2 × 10^5^ to 1 × 10^6^ cells were collected, washed with PBS, and stained with 1 mL of propidium iodide (PI)-based DNA staining solution containing RNase A and 10 μL of permeabilization solution. Samples were vortexed, incubated in the dark at room temperature for 30 min, and analyzed by flow cytometer (version 10.8.1). Cell cycle phases (G_0_/G_1_, S, G_2_/M) were quantified using FlowJo software (v10.9), and the distribution data were statistically analyzed using GraphPad Prism 8.0.

### 2.10. Immunofluorescence Staining

For immunofluorescence analysis, cells were fixed with 4% formaldehyde at room temperature for 30 min and then permeabilized with 0.5% Triton X-100 for 10 min. After removing the permeabilization solution, cells were blocked with 3% bovine serum albumin (BSA) for 30 min to reduce nonspecific antibody binding. Cells were incubated overnight at 4 °C with a primary antibody against RSF1 (Abcam, Cambridge, UK, Cat# ab109002; 1:300 dilution). Following PBS wash, a secondary antibody (Abcam, Cambridge, UK, Cat# ab150080; 1:100 dilution) was applied for 1 h at room temperature in the dark. Nuclei were counterstained with DAPI in anti-fade mounting medium and incubated for 20 min in the dark. Fluorescent images were captured using a confocal laser scanning microscope (ZEISS LSM 980 with AiryScan 2, Oberkochen, Germany).

### 2.11. Immunoblotting Analysis Assay

Total cellular proteins were extracted using enhanced RIPA lysis buffer (Boster, Wuhan, China, Cat# AR0102) following the manufacturer’s instructions. Protein concentrations were measured using a BCA assay, and equal protein amounts were separated by SDS-PAGE and transferred onto PVDF membranes.

Membranes were blocked with 5% fat-free milk in TBST for 1 h at room temperature, followed by overnight incubation at 4 °C with primary antibodies. The following primary antibodies were used: RSF1 (Abcam, Cambridge, UK, Cat# ab109002; 1:2000), GAPDH (Proteintech, Wuhan, China, Cat# 60004-1-Ig; 1:2500), Bcl-2 (Abcam, Cambridge, UK, Cat# ab182858; 1:2000), and BAX (Abcam, Cambridge, UK, Cat# ab3191; 1:1000).

After incubation with species-matched HRP-conjugated secondary antibodies, protein bands were visualized using an enhanced chemiluminescence (ECL) detection system.

### 2.12. Spontaneous ESCC Mouse Model

To induce spontaneous esophageal squamous cell carcinoma (ESCC), twenty female C57BL/6J mice (5 weeks old, 18–20 g) were purchased from Charles River and acclimatized under standard laboratory conditions for one week. Mice were randomly assigned to two groups: a control group (n = 10) and a treatment group receiving 4-Nitroquinoline-1-oxide (4-NQO; Sigma, Livonia, MI, USA, Cat# N8141) (n = 10).

The control group was provided sterile drinking water ad libitum. The 4-NQO group received 4-NQO at a concentration of 80 μg/mL in drinking water continuously for 20 weeks. This was followed by a 9-week recovery period with sterile drinking water. At the endpoint (29 weeks), all mice were euthanized. Esophageal tissues were collected, fixed in 4% paraformaldehyde, and subjected to hematoxylin and eosin (H&E) staining and immunohistochemical (IHC) analysis.

### 2.13. Xenograft Tumors Mouse Model

Female BALB/c nude mice (6 weeks old) were purchased from Charles River and randomly divided into two groups: control (n = 5) and RSF1-knockout (n = 5). EC9706 cells and RSF1-knockout EC9706 cells, both stably expressing luciferase, were used to establish subcutaneous xenograft tumors. Each mouse was inoculated with 2 × 10^6^ luciferase-labeled cells. Tumor progression was monitored weekly for 5 weeks using an in vivo bioluminescence imaging system (IVIS Spectrum, PerkinElmer, Sheldon, IA, USA). At the study endpoint, mice were euthanized, and tumors were harvested for subsequent analysis. All procedures complied with the Laboratory Animal Management Guidelines of Henan University and were approved by the Institutional Animal Care and Use Committee (IACUC Approval #: HUSOM2025-007).

### 2.14. Tissue Microarray Construction and Immunohistochemistry

A tissue microarray (TMA) containing 72 paired human esophageal cancer samples and adjacent normal tissues was obtained from Xinchao Biotechnology, (Shanghai, China).

Sections (3 μm) were prepared for histological and immunohistochemical analysis.

For H&E staining, tissues were fixed in 4% neutral buffered formalin (Beyotime, Cat# P0099) for 24 h, dehydrated, and embedded in paraffin. Sections were stained with hematoxylin and eosin (H&E) following standard protocols, and mounted with a gum-based medium.

For immunohistochemistry (IHC), paraffin sections were deparaffinized, rehydrated, and subjected to antigen retrieval. After cooling, sections were blocked with 3% bovine serum albumin (BSA) for 30 min at room temperature. The following primary antibodies were applied and incubated overnight at 4 °C: Anti-RSF1 (Abcam, Cambridge, UK, Cat# ab109002; 1:100 dilution) and Anti-Ki67 (Cell Signaling Technology, Danvers, MA, USA, Cat# 9449; 1:50 dilution). On the following day, sections were incubated with HRP-conjugated goat anti-rabbit secondary antibody (1:200 at room temperature) for 1 h. Signal detection was performed using a DAB substrate kit (Sangon Biotech, Shanghai, China, Cat# D601037). Nuclei were counterstained with hematoxylin, and slides were mounted for microscopic analysis.

### 2.15. D Label-Free Quantitative Proteome Analysis

Comparative proteomic analysis was conducted between RSF1-knockout KYSE450 cells and control cells. Total proteins were extracted, digested with trypsin, and analyzed by liquid chromatography–tandem mass spectrometry (LC-MS/MS).

Protein identification and quantification were performed using MaxQuant software (Maxquant v2.6.5.0). Differentially expressed proteins (DEPs) were defined as those with a fold change ≥ 1.5 and *p*-value ≤ 0.05. Subcellular localization predictions were made using WoLF PSORT, and protein annotations were referenced against the UniProt database. Enrichment analyses of Gene Ontology (GO) terms and KEGG pathways were conducted on the identified DEPs.

### 2.16. Statistical Analysis

All experiments were independently replicated three times to ensure reliability and reproducibility. Statistical analyses were performed using Student’s *t*-test for comparisons between groups, and one-way ANOVA with appropriate post hoc testing for multi-group comparisons. A *p*-value < 0.05 was considered statistically significant. All data were analyzed using GraphPad Prism 8.0.

## 3. Results

### 3.1. The Levels of RSF1 Are Elevated in ESCC Tissues and Mouse Models

**Figure 1:**
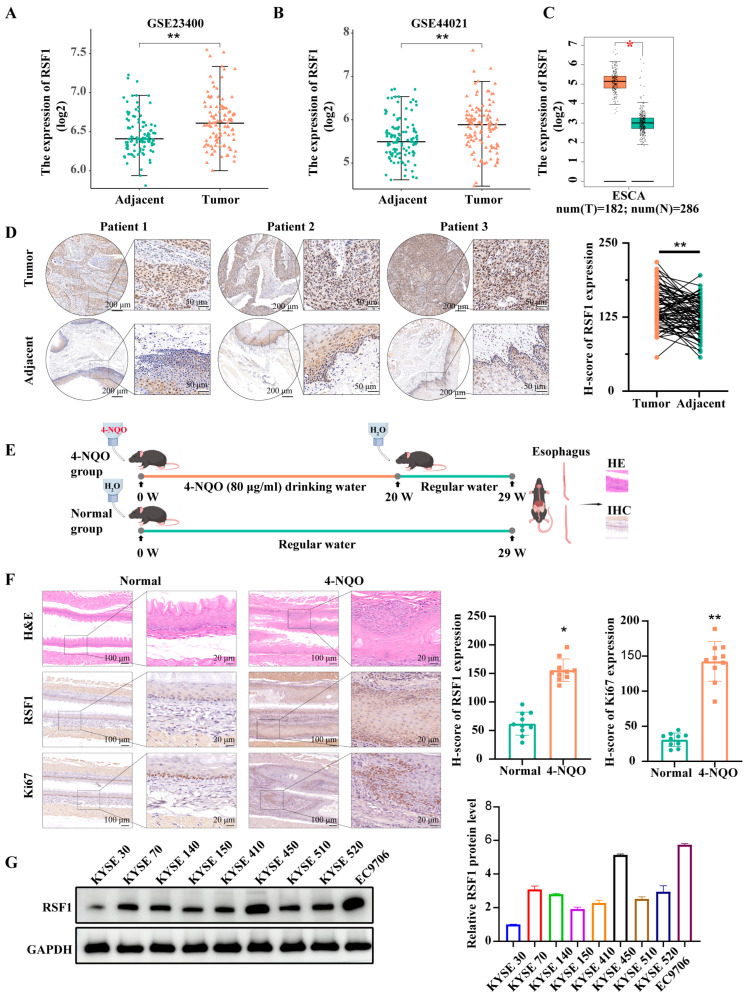
RSF1 levels are elevated in ESCC tumors. (**A**–**C**) Analysis of RSF1 mRNA expression in tumor versus adjacent non-tumor tissues using GEO datasets GSE23400 (**A**) and GSE44021 (**B**), and TCGA ESCA dataset (**C**). RSF1 expression is significantly upregulated in ESCC tumor tissues. (**D**) Representative immunohistochemistry (IHC) images showing RSF1 protein levels in tumor and adjacent tissues from three ESCC patients. Right: Quantification of RSF1 expression using H-scores (n = 60 matched samples), showing significantly higher RSF1 levels in tumor tissues. (**E**) Schematic of the 4-NQO-induced ESCC murine model. Mice were administered 4-NQO in drinking water for 20 weeks, followed by regular water until week 29, at which point esophageal tissues were harvested. (**F**) H&E staining, RSF1, and Ki-67 IHC staining of esophageal tissues from normal and 4-NQO-treated mice. Right: Quantification of RSF1 and Ki-67 H-scores showing significant upregulation in the 4-NQO group. (**G**) Western blot analysis of RSF1 protein levels in ESCC cell lines (KYSE series and EC9706). GAPDH was used as a loading control. Quantification of relative RSF1 protein levels is shown below. Data are presented as mean ± SD; * *p* < 0.05, ** *p* < 0.01.

To investigate the role of RSF1 in ESCC, we first analyzed RSF1 mRNA levels using GEO datasets GSE44021 (n  =  113) and GSE23400 (n  =  53). Both datasets revealed significantly elevated RSF1 mRNA levels in ESCC tumor tissues compared to matched adjacent non-tumor tissues (Figure 1A,B). To confirm these results, we examined RSF1 mRNA levels in a human ESCC cohort using the GEPIA database (Figure 1C), which confirmed increased RSF1 expression levels in tumors. Immunohistochemical (IHC) staining further validated these findings, with strong signals observed in tumor tissue and significantly higher H-scores in ESCC samples compared to adjacent tissues (Figure 1D). To confirm these observations in vivo, we utilized a 4-NQO-induced ESCC mouse model (Figure 1E). IHC analysis demonstrated that 4-NQO treatment led to elevated RSF1 protein level, accompanied by increased Ki-67 staining, indicating enhanced proliferation (Figure 1F). Finally, RSF1 expression was profiled across a panel of ESCC cell lines, among which, KYSE450 and EC9706 exhibited high RSF1 expression (Figure 1G). These cell lines were selected for subsequent function studies.

### 3.2. Knockout of RSF1 in RSF1-High Expression ESCC Cell Lines Suppresses Cell Proliferation, Migration, and Invasion

**Figure 2:**
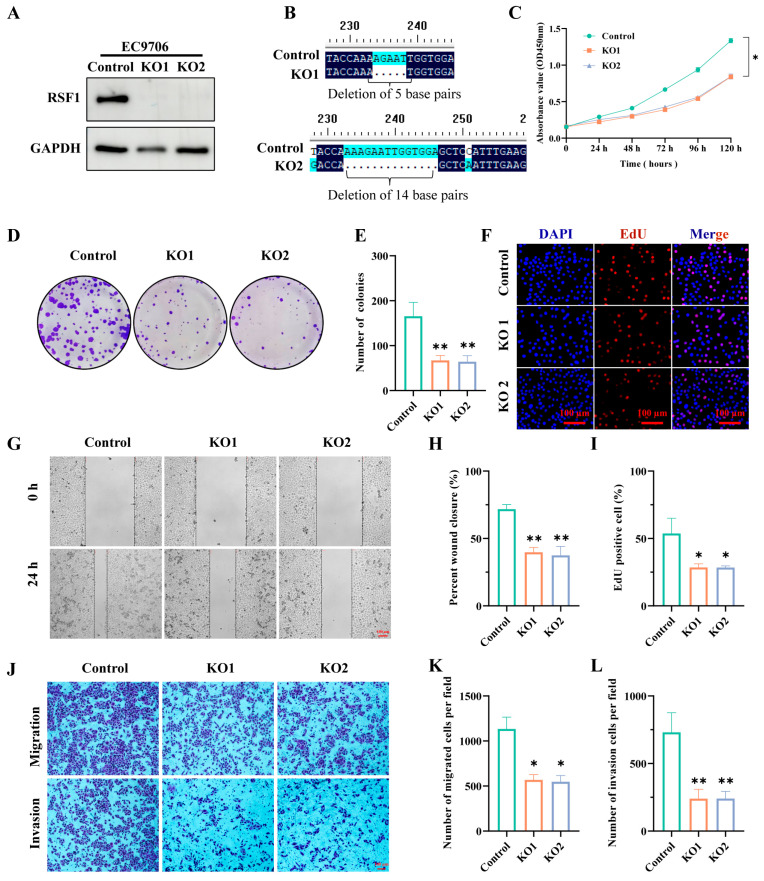
Knockout of RSF1 in RSF1-high ESCC cell line EC9706 suppresses cell proliferation, migration, and invasion. (**A**) Western blot analysis showing RSF1-knockout efficiency in EC9706 cells. Representative images are presented alongside quantification of three independent experiments. (**B**) The sequencing results show that RSF1 KO1 has a deletion of 5 base pairs, while RSF1 KO2 has a deletion of 14 base pairs. (**C**) CCK-8 assays demonstrating reduced cell proliferation in RSF1-knockout cells. (**D**–**F**) Colony formation assays showing reduced colony-forming ability post-RSF1 knockout. Representative images and quantification from three independent experiments are included. (**E**–**I**) EdU incorporation assays indicating reduced DNA synthesis following RSF1 knockout. Quantification from three independent experiments is shown on the right. (**G**,**H**) Wound-healing assays assessing cell migration and healing ability. Representative images and quantification are shown. (**J**–**L**) Transwell assays evaluating migration and invasion capabilities. Representative images and quantification are provided. Data are shown as mean ± SD. * *p* < 0.05; ** *p* < 0.01.

To elucidate the role of RSF1 in ESCC progression, we knocked out RSF1 in RSF1-high ESCC cell lines using sgRNA and examined its effects. Immunoblot analysis confirmed a significant reduction in RSF1 protein levels following sgRNA-mediated knockout (Figure 2A and Figure S2A), which were further validated by immunofluorescent staining (Figure S1). Sanger sequencing revealed base deletions and mutations in the RSF1 gene, confirming successful gene editing (Figure 2B and Figure S2B). Functional assays demonstrated that RSF1 depletion significantly impaired tumorigenic properties. CCK-8 assays showed a marked reduction in cell proliferation across both sgRNA constructs (Figure 2C and Figure S2C), while colony formation assays confirmed that RSF1 knockout diminished the cells’ ability to form colonies (Figure 2D,E and Figure S2D,E). Consistent with these results, EdU incorporation assays revealed a significant decrease in DNA synthesis in RSF1-deficient cells (Figure 2F–I and Figure S2F–I). To assess the impact on cell motility, the monolayer wound-healing assay demonstrated reduced migration in RSF1-knockout cells (Figure 2G,H and Figure S2G,H). Transwell migration and invasion assays further confirmed that RSF1 depletion significantly suppressed the cells’ migration and invasion abilities (Figure 2J–L and Figure S2J–L). Collectively, these results indicate that RSF1 is required for ESCC cell proliferation, migration, and invasion, underscoring its critical role in ESCC tumor progression.

### 3.3. RSF1 Rescued Cell Proliferation, Migration, Invasion, and Tumorigenic Activity in RSF1-KO Cells

**Figure 3:**
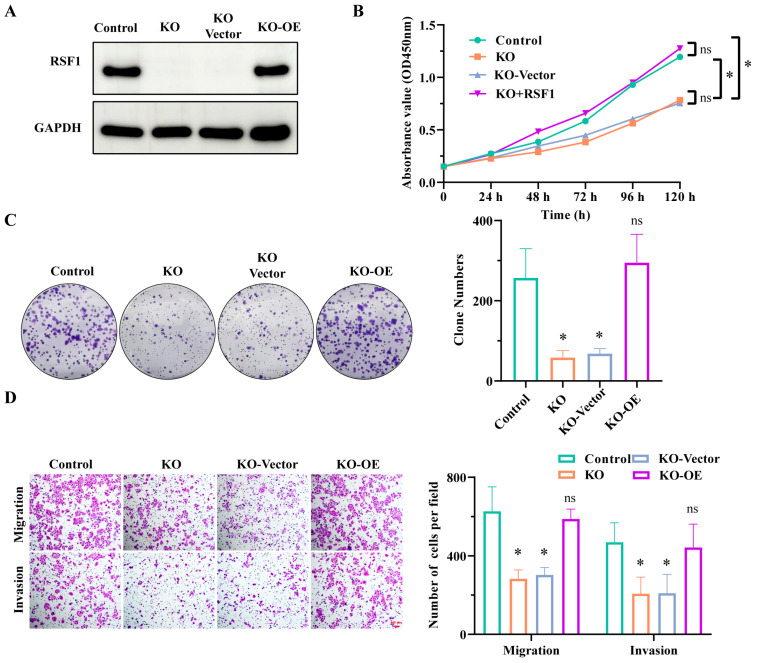
RSF1 restoration rescues the impaired tumorigenic phenotype caused by RSF1 knockout in ESCC cells. (**A**) Western blot analysis confirms RSF1 knockout by sgRNA and its restoration via RSF1 plasmid transfection. GAPDH serves as a loading control. (**B**) CCK-8 proliferation assay showing that RSF1 knockout reduces cell proliferation, which is partially rescued by RSF1 re-expression. (**C**) Colony formation assay demonstrates a significant reduction in colony numbers upon RSF1 knockout, which is rescued by RSF1 expression. Right: Quantification of colony numbers. (**D**) Transwell migration and invasion assays show reduced migratory and invasive capacities in RSF1-knockout cells, both of which are significantly restored upon RSF1 re-expression. Left: Quantification of migrated and invaded cells per field. Data are shown as mean ± SD. * *p* < 0.05.

To investigate the functional role of RSF1 overexpression in ESCC, we reintroduced RSF1 into RSF1-knockout EC9706 cells. Western blot analysis confirmed that RSF1 protein levels were restored to levels comparable to those in control cells (Figure 3A). The CCK-8 assay showed that the impaired cell proliferation observed upon RSF1 knockout was effectively rescued by RSF1 restoration (Figure 3B). Similarly, the reduced colony formation ability of RSF1-knockout cells was rescued upon RSF1 restoration (Figure 3C). Transwell migration and invasion assays further demonstrated that the suppressed motility and invasiveness resulting from RSF1 knockout were significantly recovered following RSF1 reintroduction (Figure 3D). Together, these results highlight the crucial role of RSF1 in promoting ESCC cell proliferation, migration, and invasion.

### 3.4. RSF1 Knockout Suppresses Subcutaneous Xenograft Tumor Growth In Vivo

**Figure 4:**
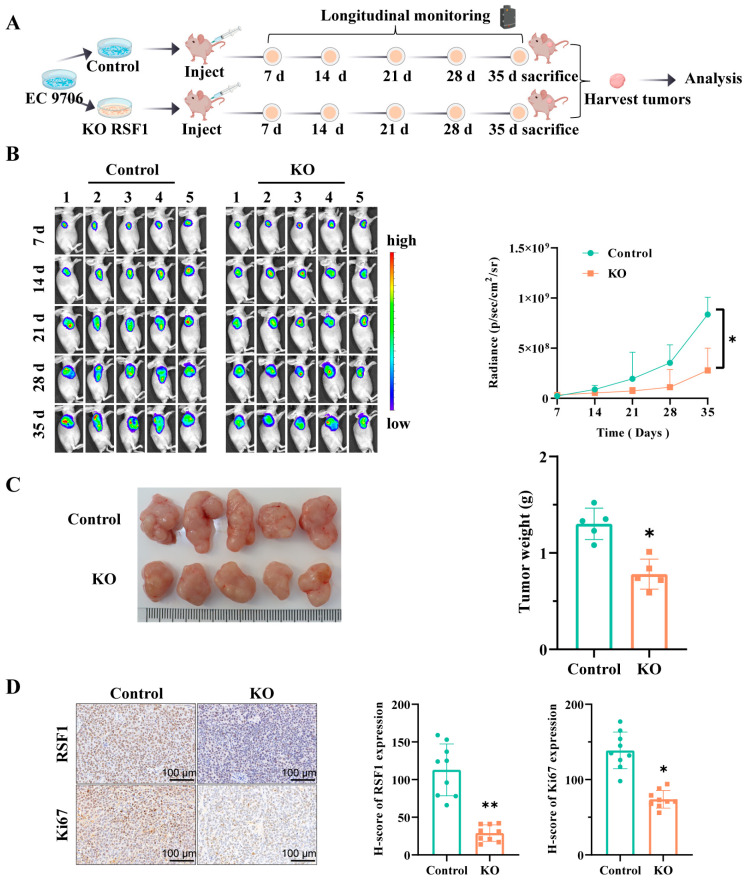
RSF1 knockout suppresses ESCC tumor growth in vivo. (**A**) Schematic for establishing mouse subcutaneous tumor xenograft models. (**B**) Bioluminescent imaging of tumor-bearing mice injected with control or RSF1-knockout EC9706 cells over a 35-day period. Tumor growth was monitored weekly, and quantification of bioluminescent signals shows significantly reduced tumor burden in the RSF1 knockout (KO) group compared to controls. (**C**) Schematic of xenograft model and representative images of tumors harvested from mice injected with control or RSF1-knockout cells. Right: Quantification of tumor weights confirms a significant reduction in tumor mass in the RSF1 KO group. (**D**) Representative IHC staining of RSF1 and Ki-67 in xenograft tumor tissues from both groups. Quantification of H-scores shows significantly decreased RSF1 and Ki-67 expression in the RSF1 KO tumors, indicating reduced proliferation. Data are shown as mean ± SD. * *p* < 0.05; ** *p* < 0.01.

To evaluate the oncogenic role of RSF1 in ESCC, we established a subcutaneous xenograft mouse model by injecting RSF1-knockout and wild-type cells (Figure 4A). The in vivo experiment revealed that RSF1 knockout significantly inhibited tumor growth over time. Bioluminescence imaging (IVIS) showed a markedly slower rate of tumor progression in the RSF1 knockout group compared to controls (Figure 4B). Consistently, tumors harvested at the study endpoint exhibited significantly lower weight in the RSF1-deficient group (Figure 4C). IHC analysis confirmed reduced RSF1 expression and decreased Ki-67 staining, indicating suppressed proliferation in RSF1 knockout tumors (Figure 4D). Collectively, these findings demonstrate that RSF1 is critical for sustaining ESCC tumor growth in vivo, and its loss significantly impairs the tumorigenic potential of high-RSF1-expressing ESCC cells.

### 3.5. RSF1 Knockout Induces Cell Cycle Arrest and Promotes Apoptosis

**Figure 5:**
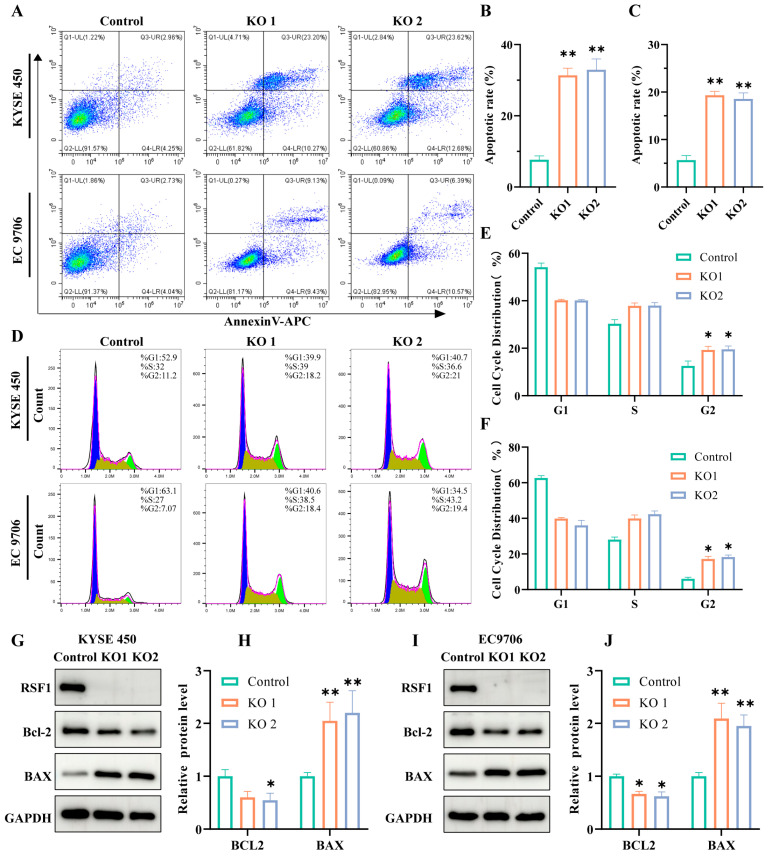
RSF1 knockout induces apoptosis, G2/M cell cycle arrest, and oxidative stress in ESCC cells. (**A**–**C**) Flow cytometry analysis of apoptosis in KYSE450 and EC9706 cells after RSF1 knockout (KO1 and KO2). Annexin V/PI staining shows significantly increased apoptotic cell populations in both KO lines compared to controls. Quantification is shown in (**B**) for KYSE450 and (**C**) for EC9706. (**D**–**F**) Cell cycle distribution assessed by PI staining and flow cytometry. RSF1 knockout increases the G2/M population and decreases S-phase cells in both KYSE450 and EC9706, indicating G2/M arrest. Quantification is shown in (**E**,**F**). (**G**–**J**) Western blot analysis of apoptosis-related proteins in KYSE450 (**G**) and EC9706 (**I**) cells. RSF1 knockout reduces anti-apoptotic Bcl-2 expression and increases pro-apoptotic BAX expression. GAPDH serves as a loading control. Quantification of the Bcl-2 and BAX expression is shown in (**H**,**J**). Data are presented as mean ± SD; * *p* < 0.05, ** *p* < 0.01.

To investigate the effects of RSF1 loss on ESCC cell fate, we performed flow cytometry analysis, which revealed that RSF1 knockout significantly promotes apoptotic cell death (Figure 5A–C). Concurrently, RSF1 knockout induced cell cycle arrest, with a marked accumulation at the G2/M phase (Figure 5D–F). Mechanistically, RSF1 depletion resulted in upregulation of the pro-apoptotic protein BAX and downregulation of the anti-apoptotic protein BCL-2, as shown by Western blot analysis (Figure 5G–J). Collectively, these results demonstrate that RSF1 knockout enforces G2/M phase arrest and activates apoptotic pathways, thereby suppressing ESCC cell proliferation.

### 3.6. Label-Free Proteomics Identified Knockout Associated DEPs, Followed by Subcellular Localization and Functional Enrichment Analysis

**Figure 6:**
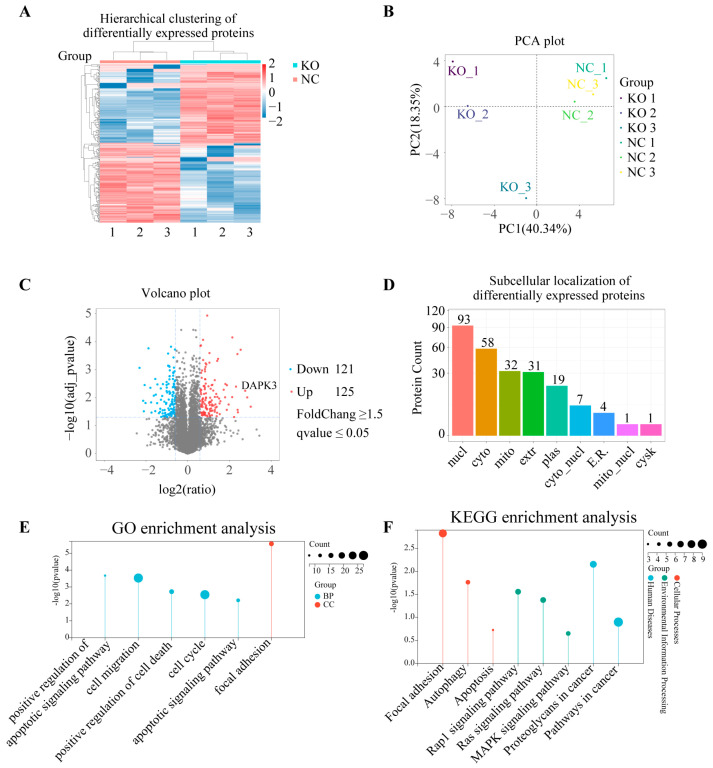
Proteomic profiling identifies RSF1-regulated pathways associated with apoptosis, cell cycle progression, and focal adhesion. (**A**) Hierarchical clustering heatmap of differentially expressed proteins in RSF1 knockout (KO) versus negative control (NC) ESCC cells, showing distinct expression patterns. (**B**) Principal Component Analysis (PCA) illustrates a clear separation between KO and NC samples, indicating reproducible global proteomic changes upon RSF1 depletion. (**C**) Volcano plot of differentially expressed proteins, highlighting 125 upregulated (red) and 121 downregulated (blue) proteins in RSF1-knockout cells (fold change ≥ 1.5, q-value ≤ 0.05). DAPK3 is highlighted as a representative significantly upregulated protein. (**D**) Subcellular localization of differentially expressed proteins based on GO cellular component analysis. The majority of affected proteins localize to the nucleus (nuc), cytoplasm (cyto), and mitochondria (mito). (**E**) Gene Ontology (GO) enrichment analysis of biological processes (BP) and cellular components (CC) shows significant involvement in apoptotic signaling, cell cycle regulation, cell death, cell migration, and focal adhesion. (**F**) KEGG enrichment analysis of differentially expressed proteins reveals significant enrichment in pathways such as focal adhesion, apoptosis, MAPK signaling, proteoglycans in cancer, and Ras signaling.

To elucidate the proteomic alterations associated with RSF1 knockout, we conducted a comparative label-free quantitative analysis between RSF1-deficient KYSE450 cells and control cells. The results revealed substantial changes in global protein profiles. Hierarchical clustering of differential protein levels (DPLs) showed distinct patterns between the KO and control groups (Figure 6A), which was further supported by Principal Component Analysis (PCA), indicating clear group separation (Figure 6B). Volcano plot analysis identified 125 significantly upregulated and 121 downregulated proteins (|log2FoldChange| ≥ 1.5, *p* ≤ 0.05) following RSF1 knockout (Figure 6C). Subcellular localization analysis of DPL indicates their primary distribution in the nucleus, cytoplasm, and mitochondria (Figure 6D). Functional enrichment analyses using GO and KEGG revealed that DPLs were significantly associated with pathways involved in cell cycle regulation, apoptotic signaling, and focal adhesion (Figure 6E,F). These findings underscore the regulatory function of RSF1 in essential cancer-related signaling pathways, indicating its involvement in modulating cell proliferation and apoptosis during ESCC progression.

## 4. Discussion

Elevated RSF1 expression has been reported across various cancer types and is often associated with poor clinical outcomes [24,25]; however, its role in esophageal squamous cell carcinoma (ESCC) has not been previously explored. In this study, we successfully knocked out the RSF1 gene using CRISPR/Cas9 technology in ESCC cell lines (KYSE450 and EC9706) and demonstrated its tumor-promoting function through both in vitro and in vivo experiments. Genetic ablation of RSF1 significantly suppressed cell proliferation, colony formation, migration, and invasion capabilities. In xenograft models using immunodeficient mice, RSF1 depletion markedly inhibited tumor growth. Reduced Ki-67 expression further confirmed RSF1′s critical role in maintaining the highly proliferative state of tumor cells.

To elucidate the oncogenic mechanisms of RSF1, we performed proteomic profiling. Results revealed that RSF1 knockout induced G2/M cell cycle arrest and robustly activated apoptosis. This pro-apoptotic phenotype correlated with upregulation of pro-apoptotic BAX, and downregulation of anti-apoptotic BCL-2, indicating RSF1 sustains tumor cell survival by regulating the mitochondrial apoptotic pathway. Additionally, proteomic analysis implicated RSF1 in focal adhesion and cell migration pathways, potentially explaining its role in promoting metastasis.

Among the DEPs identified by our proteomic analysis, DAPK3 was significantly upregulated in RSF1-knockout cells. DAPK3 (death-associated protein kinase 3) is a serine/threonine kinase known to promote apoptosis through regulation of cytoskeletal dynamics and caspase activation [26,27,28,29,30]. We hypothesize that RSF1 may transcriptionally or epigenetically repress DAPK3 expression to inhibit apoptosis, a model warranting further validation.

Although RSF1 has been implicated in breast and ovarian cancers [31,32], our findings provide the in vitro and in vivo evidence of its oncogenic role in ESCC. While we established RSF1′s overarching oncogenic function, the specific mechanisms underlying its regulation of apoptosis, particularly concerning direct targets like BAX/BCL-2 expression, remain to be elucidated. Future studies should investigate how RSF1′s chromatin remodeling function governs key gene expression and signaling pathways. Furthermore, our immunodeficient mouse model precludes assessment of RSF1′s impact on the tumor immune microenvironment. Subsequent work using immunocompetent or humanized mouse models is recommended to evaluate its immunomodulatory potential. Clinically, large-scale validation in ESCC patient cohorts is essential to determine the prognostic and therapeutic relevance of correlations between RSF1, DAPK3, Ki-67, and apoptotic markers.

Based on the aforementioned evidence, RSF1 facilitates esophageal squamous cell carcinoma (ESCC) progression by promoting tumor proliferation, migration, and invasion. Targeting RSF1 or its downstream effectors may thus offer innovative therapeutic perspectives for this aggressive malignancy.

## Data Availability

All data supporting this study are available within the article and the Supplementary Materials. Additional inquiries should be directed to the corresponding authors. The mass spectrometry proteomics data have been deposited to the ProteomeXchange Consortium (https://proteomecentral.proteomexchange.org) via the iProX partner repository [33,34] with the dataset identifier PXD067025.

## Supplementary Materials

The following supporting information can be downloaded at: https://www.mdpi.com/article/10.3390/cells14161262/s1.

## Abbreviations

The following abbreviations are used in this manuscript:

BP	Biological Processes
CC	Cellular Components
DEPs	Differentially Expressed Proteins
DPL	Differential Protein Levels
ESCC	Esophageal Squamous Cell Carcinoma
GO	Gene Ontology
PCA	Principal Component Analysis
RSF1	Remodeling and Spacing Factor 1
TMA	Tissue Microarray
4-NQO	4-Nitroquinoline N-oxide
