# Supplementary material for: Chromatin Remodeler RSF1 as an Oncogenic Driver and Therapeutic Target in Esophageal Squamous Cell Carcinoma
Source: Cells. 2025 Aug 15;14(16):1262. doi: 10.3390/cells14161262 (PMC12384802; doi:10.3390/cells14161262)
Supplement: Supplementary file 1 [file cells-14-01262-s001.zip › cells-3752154-supplementary.pdf]

Supplementary Materials:

Figure S1. Validation of RSF1 knockout by immunofluorescence.

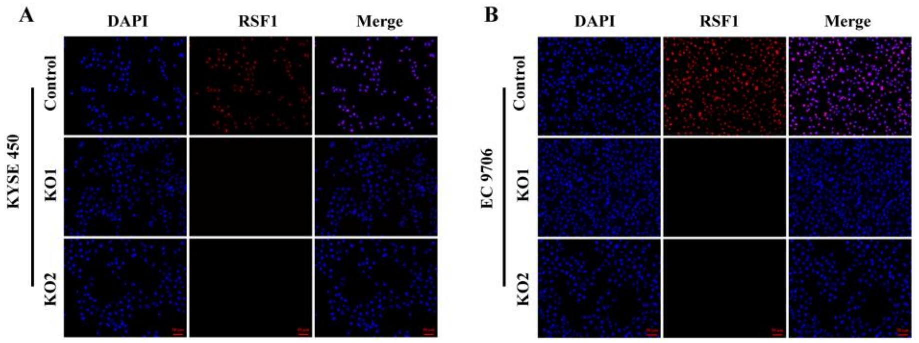

(A) Knockout of RSF1 in KYSE450 was further validated through immunofluorescence assay(B) Knockout of RSF1 in EC9706 was further validated through immunofluorescence assay.

Figure S2. Knockout of RSF1 in RSF1-high ESCC cell Line KYSE450 suppresses cell proliferation, migration, and invasion

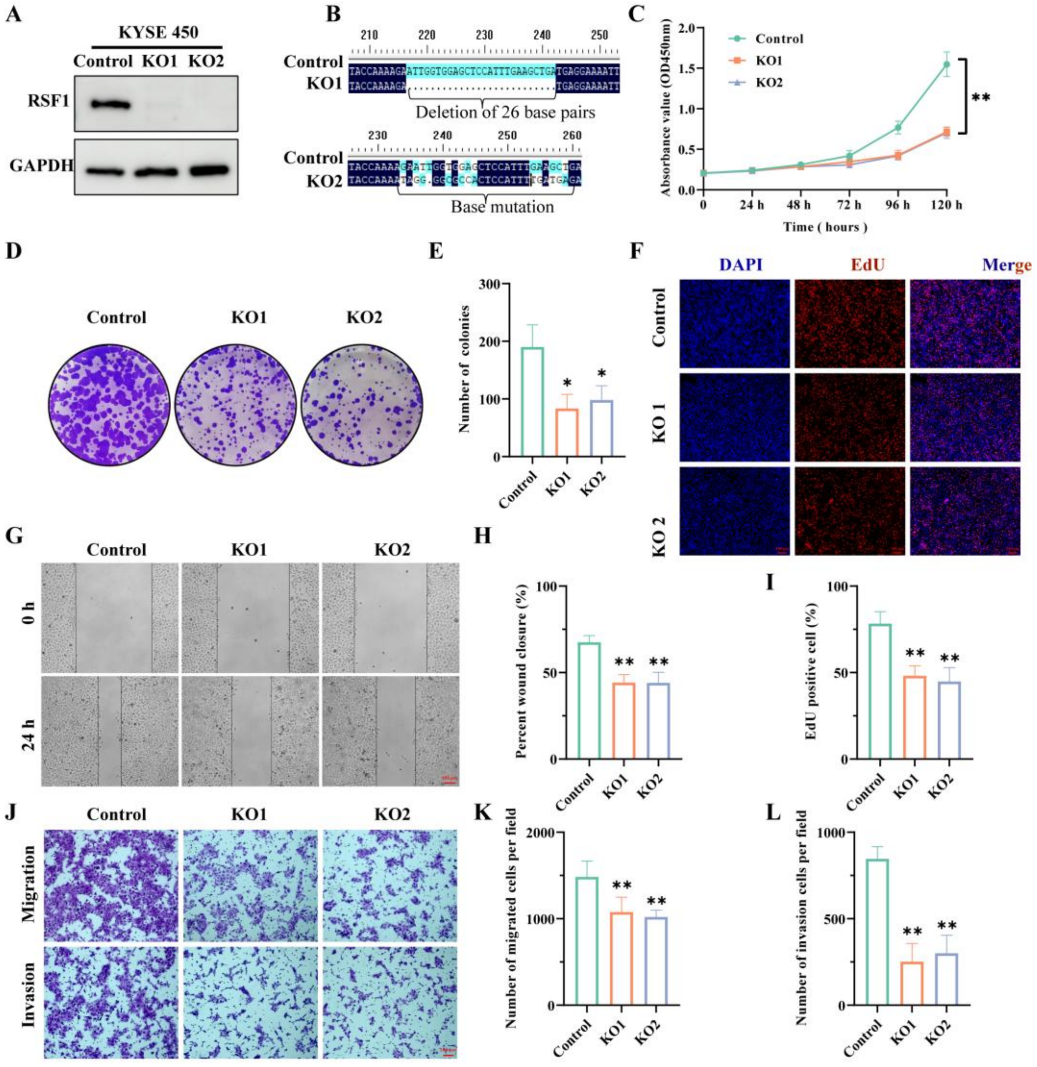

(**A**) Western blot analysis confirming effective RSF1 knockout in KYSE450 cells using two distinct sgRNAs (KO1 and KO2), with GAPDH as a loading control. (**B**) Sanger sequencing showing a 26 base pair deletion (KO1) and base mutation (KO2) in the RSF1 gene. (**C**) CCK-8 assays demonstrating reduced cell proliferation in RSF1 knockout cells. (**D-E**) Colony formation assays showing reduced colony-forming ability post-RSF1 knockout. Representative images and quantification from three independent experiments are included. (**F-I**) EdU incorporation assays indicating reduced DNA synthesis following RSF1 knockout. Quantification from three independent experiments were shown on the right. (**G-H**) Wound-healing assays assessing cell migration and healing ability. Representative images and quantification are shown. (**J-L**) Transwell assays evaluating migration and invasion capabilities. Representative images and quantification are provided. Data are presented as mean ± SD; * $p < 0.05$, ** $p < 0.01$.
